# Epistemologische Neupositionierungen. Alexander Mitscherlich zwischen „naturwissenschaftlicher Methodik“, Psychoanalyse und Psychosomatischer Medizin

**DOI:** 10.1007/s00048-021-00318-3

**Published:** 2021-11-10

**Authors:** Steffen Dörre

**Affiliations:** Institut für Geschichte der Medizin, JLU Gießen, Gießen, Deutschland

**Keywords:** Alexander Mitscherlich, Viktor von Weizsäcker, Paul Martini, Evidenzerzeugung, Kausalität, Psychoanalyse, Psychosomatische Medizin, Schulmedizin, Sozialpsychologie, Statistik, Alexander Mitscherlich, Viktor von Weizsäcker, Paul Martini, Production of evidence, Causality, Psychoanalysis, Psychosomatic medicine, Conventional medicine, Social psychology, Statistics

## Abstract

Anhand von Alexander Mitscherlichs Plädoyers für eine Psychosomatische Medizin werden dessen epistemologische Neupositionierungen in den 1940er, 1950er und 1960er Jahren untersucht. Sie eröffnen den Blick auf die Auseinandersetzung von Psychiatern, Internisten und Psychotherapeuten um valides und handlungsrelevantes Wissen in der Nachkriegszeit. Zentral war für Mitscherlich ein Krankheitsverständnis, das der Subjektivität der Patienten einen festen Platz zuwies. Damit verbunden war eine kontinuierliche Kritik an statistischen Verfahren zur Validierung von Einzelbefunden und Hypothesen. Gezeigt wird, wie anpassungsfähig Mitscherlich mit seiner Kritik an einer naturwissenschaftlich orientierten Methodik in der Medizin war, wenngleich er die ursprüngliche Stoßrichtung trotz aller Wandlungen aufrechterhielt.

## Abstract (en)

This paper examines the shifts in Alexander Mitscherlich’s epistemological position in the 1940s, 50s and 60s via his plea for psychosomatic medicine. These shifts illustrate the post-war controversy among psychiatrists, physicians, and psychotherapists about what constitutes valid and practically relevant knowledge. The subjectivity of patients is key to Mitscherlich’s concept of disease. This informs his continuous criticism of the use of statistical methods to validate individual diagnoses and hypotheses. This paper shows that Mitscherlich’s criticism of a science-based medical methodology is highly adaptable, even though, in spite of many theoretical changes and adaptations, the main thrust of his approach remains consistent.

## Problemaufriss

Nach dem Zweiten Weltkrieg etablierte sich die Psychosomatik^1^ in der Bundesrepublik Deutschland als eigenständige medizinische Disziplin. Im Vorfeld und auch während dieses Institutionalisierungsprozesses spielten Debatten zwischen Internisten, Psychiatern und Psychosomatikern über das Menschenbild in der Medizin und eine angemessene Methodologie eine wichtige Rolle. In den oftmals harschen Auseinandersetzungen verfeinerten die Vertreter der jeweiligen Disziplinen ihre Einwände an den konkurrierenden Ansätzen. Sie schärften aber auch ihre Konzeptionen von relevantem und validem Wissen. Im vorliegenden Text wird die Kritik an der naturwissenschaftlich orientierten Methodik in der Medizin stellvertretend am Beispiel von Alexander Mitscherlich (1908–1982) analysiert. Mitscherlich war eine Leitfigur der institutionellen Entwicklung von Psychosomatik und Psychoanalyse in Westdeutschland und prägte deren Entwicklung über mehrere Jahrzehnte als Publizist, klinischer Praktiker, Ordinarius, Dokumentarist des Nürnberger Ärzteprozesses und öffentlicher Intellektueller maßgeblich mit (Dehli 2007; Freimüller 2007; Freimüller 2019); Hoyer 2008; Zitat Müller & Ricken 2004: 219). Für ihn versprach die Psychosomatische Medizin eine Heilkunst, die sich nicht nur auf leicht zu lokalisierende Funktionsstörungen einzelner Organe konzentrierte, sondern danach strebte, den ganzen Menschen zu erfassen, zu verstehen und zu therapieren (Roelcke 2020: 49).

Im Folgenden untersuche ich Mitscherlichs epistemologische Neupositionierungen, beginnend mit seiner Stellungnahme auf dem Kongress der Deutschen Gesellschaft für Innere Medizin (DGIM) im Jahr 1949. Nach einem kürzeren Einschub zur allgemeinen Lage der Psychotherapie in den 1950er Jahren konzentriere ich mich auf Mitscherlichs Aussagen aus den späten 1950er und den 1960er Jahren. Zu dieser Zeit formulierte Alexander Mitscherlich seine Kriterien für valides Wissen noch einmal genauer und unterfütterte seine Kritik am Kausalitätsdenken in der medizinischen Forschung mit sozialpsychologischen und psychoanalytischen Argumenten.

## Der Kongress der Deutschen Gesellschaft für Innere Medizin 1949

### Plädoyers für eine Psychosomatische Medizin

Ein erster wichtiger Kristallisationspunkt der Auseinandersetzungen über den Status der Psychosomatischen Medizin in der westdeutschen Nachkriegsmedizin war der Kongress der Deutschen Gesellschaft für Innere Medizin im April 1949.^2^ Der gesamte erste Verhandlungstag war diesem Thema gewidmet. Curt Oehme (1883–1963), Tagungspräsident und der Psychosomatischen Medizin wohlgesinnt, umriss in seiner Eröffnungsansprache den Deutungsrahmen, in dem er die nachfolgenden Stellungnahmen sah: In einer Situation, in der die „verbrecherische[n] Vorkommnisse“ der Nazizeit auch auf das „Menschenbild der Medizin“ zurückzuführen seien, in der man vom internationalen Stand von Theorie und Forschung immer noch abgeschottet sei, in der die „täglichen Erlebnisse“ einem vor Augen führten, „daß allein mit der objektivierenden Anschauungsweise der Naturwissenschaft die Heilkunde die Bedürfnisse der Kranken praktisch sehr oft nicht decken kann“, habe man die „Richtigkeit und Brauchbarkeit“ des Menschenbildes in der Medizin zu prüfen. Zwar hätte sich die „streng naturwissenschaftliche Gesinnung“ der Medizin aufgelockert und längst seien auch „die Einstellungen und Irrtümer“ bekannt, die daraus resultierten, „wenn die Innensphäre des Menschen, die oft besonders mit seiner sozialen verknüpft ist, zu stark hinter den biologischen Außenaspekt zurückgestellt“ werde, doch hätten sich diese Erkenntnisse bislang kaum auf die Reichweite der Rationalisierungen und naturwissenschaftlichen Erklärungen ausgewirkt. Auch wenn Oehme viele seiner Gedanken als offene Fragen formulierte, war unmissverständlich, was er sich von Psychotherapie und Tiefenpsychologie – die er als „moderne“ Ausformung einer älteren Psychosomatik verstand – versprach: einen „Anstoß zur Wandlung des ärztlichen Menschenbildes und Menschenverständnisses“ (Oehme 1949: 3–8, Zitate: 3, 4, 6, 8).

Nach Oehme betrat Viktor von Weizsäcker (1886–1957), zu diesem Zeitpunkt Ordinarius für Allgemeine Klinische Medizin an der Ruprecht-Karls-Universität in Heidelberg, die Bühne. Er hatte schon seit vielen Jahren einen Paradigmenwechsel in der Medizin durch die Einbeziehung des menschlichen Subjekts gefordert und in scharfem Kontrast zur naturwissenschaftlichen Medizin eine „anthropologische Medizin“ entworfen (Geisthövel 2019). Kein anderer deutscher Arzt, so die Einschätzung des Wissenschaftshistorikers Michael Hagner, formulierte im 20. Jahrhundert „so beharrlich und kompromißlos aus der wissenschaftlichen Medizin heraus eine ähnlich einflußreiche Kritik derselben“ (Hagner 2006: 336). Seine Ausführungen auf dem DGIM-Kongress begann Weizsäcker mit den von der Medizin enttäuschten Patienten. Seiner Ansicht nach stand die „gesamte Medizin […] teils durch Kritik von außen, teils durch Selbstkritik, teils durch die Einwirkung psychologischer Denkweise und Methode“ vor großen Herausforderungen. Mittlerweile veränderten die Erkenntnisse über das Zusammenspiel von Leib und Seele nicht mehr nur, wie es noch Sigmund Freud (1856–1939) konzipiert habe, den mit den Hysterien, Phobien und Zwangsneurosen befassten Teil der Psychiatrie. Die Psychosomatische Medizin präsentierte Weizsäcker nicht lediglich als eine ergänzende Perspektive – sie sollte mehr sein als „die fortgesetzte Anwendung der Laboratoriumsapparatur auf die zum Objekt versteinerten Affekte der Insassen eines Spitals“. Verstehe man sie anthropologisch statt naturwissenschaftlich, habe die Psychosomatische Medizin das Potenzial, die Medizin umfänglich zu reformieren.^3^ Viktor von Weizsäcker entwarf ein ambitioniertes Programm: Die anthropologische Psychosomatik sollte erstens konsequent tiefenpsychologisch ausgerichtet sein, zweitens nicht nur individualpsychologische, sondern auch kollektiv- und massenpsychologische Erkenntnisse liefern und drittens „die naturwissenschaftliche Biologie (allmählich oder revolutionär) veränder[n]“.^4^ Den anwesenden Medizinern stellte er dabei in Aussicht, „daß mit psychosomatischer Medizin die Heilkunde und -kunst nicht noch komplizierter, sondern daß sie wieder einfacher wird“ (Weizsäcker 1949: 13–18, 23 f., Zitate ebd.).

Weizsäcker hatte erwartet, dass Alexander Mitscherlich, der bei ihm promoviert worden war und habilitiert hatte, in seinem Vortrag inhaltlich „vor allem auf die Tuberkulose eingehen“ würde, da er diese „seit geraumer Zeit studiert[e]“ (Weizsäcker an Jores, 21.02.^1949^). Doch Mitscherlich ließ es nicht dabei bewenden. Er fragte stattdessen, wie man auf die von Weizsäcker angesprochene Erweiterung des Wissens über leib-seelische Zusammenhänge reagieren könne. Seine Hoffnungen richteten sich auf eine „dynamische Psychologie“, die „den ganzen inneren Reichtum [der] biographischen Situationen“ erfasse. Krankheiten ließen sich mit ihr als Zeichen oder Symbole für psychische Konflikte, als adaptive „Fehlleistung“ des Subjekts, als „Schicksalsvollzug“ erklären. Weil sie nicht nur „ihrer jeweiligen Besonderheit – als Lungen‑, Herz-, Magenkrankheit usw. – sondern durch den Augenblick ihres Ausbrechens einen Bezug zu der Art und Weise, wie dieses Leben subjektiv geführt wurde“ beinhalteten, warb er darum, die Lebensgeschichte zum Ausgangspunkt des Diagnose- und Behandlungsprozesses zu machen.^5^ „Methodisches Kernstück der psychosomatischen Medizin“ war für ihn, so gab er auf der DGIM-Tagung zu Protokoll, „von der manifesten Krankheit zurück zum Sinn des Geschehens zu fragen“ (Mitscherlich 1949: 24–27, 32).^6^

Arthur Jores (1901–1982), Professor für Innere Medizin in Hamburg, der von Oehme gebeten worden war, einen kurzen Kommentar zu den beiden Vorträgen von Mitscherlich und Weizsäcker abzugeben (Jores an Weizsäcker, 15.02.1949), beschränkte sich in seinem Koreferat auf wenige Anmerkungen. Für ihn war die Psychosomatik nicht bloß „eine neue Sonderdisziplin, sondern eine grundlegend neue Anschauungsweise und Auffassung der Krankheit und des kranken Menschen“, ja die „Medizin der Zukunft“ (Jores 1949: 57 f.). So ähnlich hatte dies auch schon bei den ersten beiden Rednern geklungen. Weizsäcker hatte selbstsicher davon gesprochen, dass der einzelne Mensch, die Medizin und die gesamte Gesellschaft „an Psychosomatik […] genesen“ könne. Eine „recht verstandene psychosomatische Medizin“ habe einen „umstürzenden Charakter“ (Weizsäcker 1949: 17, 21).^7^ Mitscherlich teilte diesen Anspruch, wenngleich er ihn auch stärker als Weizsäcker auf die Medizin beschränkte. Auch für ihn war die Psychosomatische Medizin ein grundlegender Perspektivenwechsel, dessen Grenzen erst nach „ausgedehntere[n] kasuistische[n] Erfahrungen“ absehbar sein würden, der aber jetzt schon ein Ferment für „einen Stilwandel der ärztlichen Kunst“ bereitstelle (Mitscherlich 1949: 32 f., 40; Aussprache 1949: 77).^8^ Damit gingen die drei Genannten deutlich über das von Oehme entworfene Szenario hinaus – dieser hatte die Psychosomatische Medizin in seinen einleitenden Worten nicht als revolutionären Akt, sondern lediglich als eine „Art Zusammenschau“ von Biologie und dem „Innenwesen des Menschen“ vorgestellt (Oehme 1949: 12).

Seine kurze Redezeit nutzte Jores dazu, um vor „Dilettantismus“ zu warnen. Eine „gute Kenntnis sowohl im Bereich des Somatischen als auch des Psychischen“ sei unabdingbar. Deshalb könne man auch nicht auf die „Mitarbeit der Psychologen, Psychiater und Psychotherapeuten“ verzichten (Jores 1949: 57 f.). Dies leitete zum Redebeitrag des Psychotherapeuten Joseph Meinertz (1877–1968) über. Meinertz war kein Ordinarius, sondern zunächst Leitender Arzt, dann zwischen 1926 und 1944 Direktor der Inneren Abteilung im Städtischen Krankenhaus Worms gewesen und blieb auch aufgrund seines Alters im weiteren Verlauf für die Debatte randständig. Doch auf dem DGIM-Kongress wurde ihm ein prominentes Forum zugestanden. An Weizsäckers und Mitscherlichs Vorträgen gefiel ihm, dass hier ein direkter Sinnzusammenhang hergestellt wurde: „daß auch das organische Symptom etwas ‚meinen‘ kann, indem es der symbolische Ausdruck einer bestimmten seelischen Situation ist“. Denn auch für ihn war mittlerweile die „Grenze zwischen ‚organisch‘ und ‚funktionell‘ so fließend geworden“, dass nicht mehr daran zu zweifeln war, dass organische Vorgänge „den Sinn symbolisch vor Augen führen“ (Meinertz 1949: 62).

Weizsäcker, Mitscherlich, Jores und Meinertz einte der Verweis auf den Nutzen der Psychoanalyse beziehungsweise der Tiefenpsychologie. Auch wenn sie sie unterschiedlich interpretierten, verwiesen sie an zentralen Stellen ihrer Darlegungen auf die Erkenntnisse Sigmund Freuds. Für Mitscherlich hatte Freud gar „eine copernikanische Wendung“ eingeleitet (Aussprache 1949: 80). An dieser Stelle des Argumentationsgangs ist es zunächst aber wichtiger festzuhalten, wie selbstbewusst die Protagonisten auf dem DGIM-Kongress auftraten. Die Psychosomatische Medizin war ihrer Meinung nach nicht nur praxistauglich, sondern auch zukunftsweisend.

### Relevant! Aber auch valide?

Die Existenz psychosomatischer Prozesse stand für Weizsäcker, Mitscherlich, Jores und Meinertz außer Frage. Sie hielten sie mittlerweile für so offenkundig, dass sie kaum noch belegt werden mussten. Viktor von Weizsäcker sagte es ganz direkt: Es komme nicht mehr darauf an, „Beispiele und Argumente dafür zu bringen, daß in Pathologie und Therapie auch Seelisches mitspricht“ (Weizsäcker 1949: 13 f.). Mitscherlich zitierte als Beleg den anwesenden Bonner Internisten Paul Martini (1889–1964) mit dessen Aussage, es könne „tatsächlich und grundsätzlich beim Menschen kein einziger Fall von Krankheit und ihrer Heilung erdacht werden, bei dem Zusammenordnung von Geist, Seele und Leib keine Rolle spielen würde“ (Mitscherlich 1949: 33; Zitat aus Martini 1948: 346).

Zur Debatte stand aber die Validität des Wissens. An ihr würde über die Wissenschaftlichkeit des psychosomatischen Ansatzes entschieden werden. Diesbezüglich gaben die vier genannten Redner bereitwillig zu, dass es an Forschung fehle, dass es noch viele Hindernisse und Probleme gebe und dass man an psychosomatische Aussagen die gleichen Ansprüche zu stellen habe wie an die naturwissenschaftliche Medizin. So forderte beispielsweise Viktor von Weizsäcker, dass „das Seelisch-Unbewußte […] mit gleicher Akkuratesse und Kritik erforscht werde […], wie der Körpervorgang“; interessant sei dabei jedoch nur, „was empirisch untersuchbar und entscheidbar ist“. Weizsäcker setzte große Hoffnungen in eine noch zu etablierende experimentelle Psychosomatik, die neben der „treulichen, redlichen Beobachtung“ auch die Motive und Ziele der Forscher „wachsam und mißtrauisch“ im Auge behalte. Er schrieb der psychosomatischen Forschung als dringlichste Aufgabe die „Entzifferung der Organsprache“ und die „Übersetzung in das der Seele verständliche Wort“ ins Stammbuch (Weizsäcker 1949: 14–19). Alexander Mitscherlich bemängelte das Fehlen einer klinischen Psychologie, die „an Subtilität und methodischer Erprobtheit [… mit] den biologischen Kenntnissen schon Schritt halten könnte“. Er verlangte die Sammlung kasuistischer und experimenteller Erfahrungen und die Katalogisierung psychophysiologischer Simultanabläufe (Mitscherlich 1949: 26, 29).

Angesichts der defizitären Forschungslage kam der Erfahrung in der Klinik eine besondere Rolle zu. Weizsäcker war überzeugt, dass diese auch jetzt schon ausreichend belege, dass Krankheiten „auf dem Höhepunkt einer biographischen Krise“ einträten. Durch die „täglichen Beobachtungen“ in der Klinik sei offenkundig, „daß statt eines in der Liebe, in der Fortpflanzung, in der Arbeit, im Geiste ungelebten Lebens ein körperliches Symptom auftritt“ (Weizsäcker 1949: 20). Mitscherlich diente die durch eine psychologische Intervention am Kranken hervorgerufene augenscheinliche Wirkung der Therapie als Beleg für die Richtigkeit seiner Annahmen: Schnell verliere sich die „Bedrohlichkeit des Krankheitsbildes […], wenn der Patient zur offenen Annahme seiner Situation und zu einer unverhohlenen Äußerung seiner Gefühle gebracht wird“ (Mitscherlich 1949: 29). Die Erfahrung der Klinik sei so überwältigend, dass es vorerst ausreiche, dies als Tatsache zur Kenntnis zu nehmen, auch wenn man die Funktionszusammenhänge noch nicht verstehe. Joseph Meinertz benutzte hierfür sogar explizit den Begriff einer „erlebnismäßigen Evidenz“ (Meinertz 1949: 61).

### Kritik an der naturwissenschaftlich orientierten Methodik in der Medizin

Dass Belege für die Unumstößlichkeit ihrer Beweisführungen noch fehlten, schien den vier genannten Medizinern kein größeres Problem darzustellen. Zum einen forderten sie ja selbst lautstark weitere Forschung, zum anderen begriffen sie die Kriterien für valides Wissen in der naturwissenschaftlichen Medizin als Scheinlösung. Oehme hatte sich schon in seiner Eröffnungsansprache kritisch zu den Auswirkungen der „naturwissenschaftliche[n] Methodik“ in der Medizin geäußert und „eine schärfere und einheitlichere, begriffliche und denkerische Durchbildung der Prinzipien“ eingefordert (Oehme 1949: 5). Weizsäcker betonte, dass Wissenschaft heute – und damit meinte er wenige Jahre nach den nationalsozialistischen Medizinverbrechen – lediglich „eine ganz bestimmte menschliche Haltung unter anderen“ sei, mit der man „Gutes und Böses tun kann“. Die Vertreter der naturwissenschaftlichen Erkenntnisweise hätten sich zu fragen, ob die Art ihres Erkenntnisstrebens und ihrer Wissenserzeugung per se „wirklich gut“ sei. „Wissenschaftliche Objektivität“ sei nicht mehr sakrosankt, „wenn Naturwissenschaft eine Form der Naturbeherrschung [und] Objektivität eine Art, die Subjektivität zu vernichten ist“ (Weizsäcker 1949: 14 f.).^9^ Auch Mitscherlich ließ die Beobachtungen, die er als Berichterstatter über den Nürnberger Ärzteprozess (1946/1947) gemacht hatte, in seine Argumentation einfließen. Hier war er schrittweise zu der Überzeugung gelangt, dass die „Organmedizin“ zwangsläufig eine inhumane Einstellung zum Patienten hervorbringe. Die Medizinverbrechen waren für ihn nicht Ausdruck individueller ärztlicher Skrupellosigkeit, sondern die Bankrotterklärung einer medizinischen Wissenschaft, die sich nicht mehr am individuellen Wohl des Patienten, sondern an abstrakten Entitäten orientierte. Im Nationalsozialismus habe man erleben müssen, welches zerstörerisches Potenzial eine einseitig an den Naturwissenschaften orientierte Medizin beinhalte. Sie hatte nicht mehr Menschen behandelt, sondern Patienten objektiviert, zu bloßen Symptomträgern degradiert und schließlich vernichtet (Mitscherlich 1946/1983a; Mitscherlich 1947/1983b: 160; Mitscherlich 1948/1983c: 413, 415 sowie Freimüller 2007: 45 f.; Roelcke 2020: 52).

Während Weizsäcker mit seiner Kritik an der naturwissenschaftlichen Medizin auf der philosophischen Ebene verharrte, wurde Mitscherlich konkreter. Er bezeichnete „Denkgewohnheiten, die im physikalischen und physiologischen Experiment wurzeln“ als ungenügend und forderte, dass sich „die psychosomatische Forschung von den fragwürdigen Methoden statistisch nachgewiesener Erfolge fernhalten und statt dessen in der anthropologischen Verantwortung bleiben“ solle. In diesem Zusammenhang wies er auf ganz „grundsätzliche Differenzen in der Wissenschaftsmethodik“ hin. Er gestand der von Forschern wie Martini geforderten „strenge[n] Kausalanalyse“ zwar „ihren Sinn und ihren Platz“ in der psychosomatischen Krankheitsbetrachtung zu, sah sie aber bei der Beantwortung der Frage, welchen Sinn die Krankheit habe, an die Grenzen ihrer Brauchbarkeit stoßen. Das Subjekt sei selbst „kausalanalytisch nicht adäquat faßbar“ (Mitscherlich 1949: 27, 33 f., 40).

Die Fürsprecher der psychosomatischen Ansätze fragten also nicht, wie sich die in der Medizin etablierten Methoden, valides Wissen zu bezeugen, in angepasster Form auf die Beschreibung psychosomatischer Zusammenhänge übertragen ließen. Auch ging es ihnen nicht um veränderte Standards innerhalb eines gegebenen allgemeinen Bezugssystems. Vielmehr waren sie der Überzeugung, dass sie für die neue Wissens- und Wissenschaftsform gänzlich andere Kriterien benötigten. Dies dürfte die Zuhörer nicht allzu sehr überrascht haben. Bereits zuvor war das „kausale Denken“ bei der Durchdringung des Psychischen und Psychosomatischen als unbrauchbar bezeichnet worden.^10^ Alexander Mitscherlich hatte beispielsweise schon in seiner zwischen 1943 und 1945 entstandenen Schrift über *Freiheit und Unfreiheit in der Krankheit* in ähnlicher Weise argumentiert. So hatte er – aus bewusst erkenntnistheoretischer Perspektive –11 Bestrebungen gegeißelt, die Pathologie ausschließlich zu „einer Wissenschaft meßbarer Körpervorgänge zu machen“. Die „Verquickung“ von Reduktionsverfahren mit Logik und empirischer Kontrolle habe, so seine damalige Interpretation, in den modernen Naturwissenschaften zwar die „systematische und deduktive Durchforschung der Welt“ ermöglicht, doch hätten „Kausalanalyse“ und „rationale[r] Empirismus der angewandten Naturwissenschaften“ die Trennung von physischen und psychischen Faktoren begünstigt und betoniert. Mitscherlich machte also auf dem DGIM-Kongress nicht zum ersten Mal die naturwissenschaftliche Erkenntnismethode dafür verantwortlich, dass psychische Leistungen ausschließlich aus der Beobachtung und Messung von Organleistungen und materiellen Vorgängen erklärt würden (Mitscherlich 1946/1977: 9, 25 f., 29).

Im gleichen Atemzug, in dem die Kausalgesetze aus der mechanistischen Biologie auf die Medizin und die psychischen Vorgänge übertragen worden seien, habe man einen gewichtigen Teil der menschlichen Existenz aus der Heilkunde ausgeschlossen. Das Streben nach allgemeingültigen wissenschaftlichen Aussagen habe in reduktionistischer Weise den eigentlichen Wesenskern des Untersuchungsgegenstands „vernichtet“. Wolle man zu einer den Menschen gerechteren Anschauungsweise kommen, sei zu klären, „welcher Art der Rest der nicht kausal-analytisch zu bewältigenden Phänomene ist, und ob er aus der Wissenschaft auszuscheiden hat oder nicht, ob über ihn wissenschaftliche Aussagen überhaupt möglich sind oder nicht“. Habe man denn zwangsläufig Wissenschaft mit Kausalanalyse zu identifizieren? Wäre es nicht hilfreicher, „zu einem ‚Pluralismus‘ der Betrachtungsweisen [zu] gelangen“, statt das Projekt, dem Subjekt wissenschaftlich gerecht zu werden, aufzugeben? Ein „additives Verfahren“, eine lediglich kleine „Modifikation der analytischen Methode des Begreifens“ werde nicht ausreichen. Das Subjekt müsse „im Ansatz der methodischen Anschauung miterfaßt werden“ (Mitscherlich 1946/1977: 30 f., 35 f., 39 f.).

Mitscherlich plädierte hier nicht allein für die Pluralität von Wissen, sondern verwies auch eindringlich auf die Unvereinbarkeit der Standpunkte. Der Gültigkeitsbereich einer jeden Forschungsmethode höre dort auf, „wo die Erscheinungen einen grundsätzlich anderen Charakter annehmen“. Wolle man das Wesen psychosomatischer Relationen verstehen, habe man sich der unfruchtbaren Kausalanalyse zu entziehen. Jedes Subjekt habe schließlich seine eigenen Gesetze, die sich nur begreifen ließen, wenn man sich auf selbiges einlasse. Die „naturwissenschaftliche Medizin“ habe ihre Erkenntnisgrenze erreicht und müsse sich angesichts dieser Tatsache eingestehen, dass die Wahl ihrer Methode lediglich eine „Stilfrage“ sei (Mitscherlich 1946/1977: 44).

### Zwei nichtkompatible Wissensformen

Alexander Mitscherlich hob in der den Vorträgen folgenden Aussprache hervor, dass er erheblichen Widerstand erwartet hatte. Immerhin hatten sich die Vertreter psychosomatischer Ansätze wiederholter Vorwürfe von Unwissenschaftlichkeit und Mystizismus erwehren müssen.^12^ Angesichts dieser schwierigen Ausgangslage hatte Mitscherlich in seiner Rede Einwände vorweggenommen und war prophylaktisch zum Angriff übergegangen. Zudem hatte er das verfügbare Instrumentarium zur Etablierung und Plausibilisierung neuer Ansätze in der Medizin ausgereizt: Er bezog sich auf die „fortschrittliche“ Forschung im Ausland, allen voran den USA, und die ökonomische Effizienz psychosomatischer Behandlungen; er beschwor den therapeutischen Nutzen psychosomatischer Betrachtungsweisen, verwies auf das bislang zusammengetragene empirische Erfahrungsgut, auf die Fülle aussagekräftiger Fallgeschichten und auf die große Menge verfügbarer Forschungsliteratur. Ferner hatte er die anwesenden Internisten geschickt mit Beispielen aus dem Bereich der Lungen‑, Herz- und Magenkrankheiten umworben. Die Psychiater griff er hingegen direkt an. Er unterließ jeglichen Versuch, nach Überschneidungen zwischen Psychosomatischer Medizin und Psychiatrie zu suchen oder um Anerkennung bei den mit ihm auf dem Podium sitzenden Psychiatern zu werben.^13^ Vielmehr grenzte er sich bewusst gegen sie ab und verwies – ein wohl kalkulierter Affront – auf die weitreichenden und hilfreichen Erkenntnisse der psychoanalytischen Forschung und die Wirkung einer „geduldig methodisch exakt geführten psychoanalytischen Behandlung“ (Mitscherlich 1949: 39).^14^

Das Verhältnis der Vertreter psychodynamischer Ansätze zu den Psychiatern war schon seit Langem belastet. Auch in der unmittelbaren Nachkriegszeit wurde die Psychotherapie nicht nur als Reformkraft begrüßt, sondern auch als eine bloße „Weltmode“ abgetan (Stöckel 2013: 320 f.). Alexander Mitscherlich hatte 1946 in einem Schreiben an den Dekan der Medizinischen Fakultät Heidelberg seine Sicht auf die Beziehungen der beiden Berufsgruppen zueinander in klare Worte gefasst:

Es hieße einen Tatbestand verschleiern, wenn man nicht offen aussprechen würde, daß die psychiatrische Forschung während des vergangenen halben Jahrhunderts an der Fragestellung in der Psychologie des Unbewußten entweder vorbeigegangen ist oder aber sie offen bekämpft hat. Eine Förderung hat sie in keinem einzigen Fall erfahren! Die Psychotherapie als Anwendung der Psychologie des Unbewußten hat sich ohne jeden Zusammenhang mit der Psychiatrie entwickelt (Mitscherlich an Engelking, 03.05.1946).

Auf dem DGIM-Kongress des Jahres 1949 betonte er, dass die „um die psychosomatische Problematik ringenden Ärzte“ als „Fremdlinge, Neuerer, gar Schwärmer mißtrauisch an den Pforten der Kliniken und Universitäten […] gehalten“ würden (Mitscherlich 1949: 40). Ähnlich hatte sich auch Viktor von Weizsäcker positioniert. Er sprach von dem „Verhängnis“,

daß die Psychoanalyse Sigmund Freuds zuerst mißhandelt, dann nicht selbst erprobt und dann verfälscht worden ist, und dies leider besonders von seiten der reichsdeutschen Psychiatrie, obwohl die Psychoanalyse vor allem die tiefenpsychologische und wissenschaftliche Potenz ist, kraft welcher eine Psychosomatik aufgebaut werden muß (Weizsäcker 1949: 17).

Dies galt Weizsäcker zufolge nicht nur für die Vergangenheit. Eine „unentwegt fortgesetzte Ignorierung der Psychosomatik“ sei auch jetzt noch zu beklagen (Weizsäcker 1949: 20).

Der Widerspruch seitens der anwesenden Psychiater kam folglich nicht unerwartet.^15^ Die Vorträge von Mitscherlich und Weizsäcker zielten auch gar nicht darauf, deren Zweifel an den tiefenpsychologischen und psychosomatischen Beweisführungen zu zerstreuen. Zudem waren die Psychiater direkt angegriffen worden und fühlten sich wahrscheinlich auch von der demonstrativ zur Schau getragenen Selbstgewissheit und den erhobenen Ansprüchen herausgefordert. Schließlich war die Kritik an den mechanistischen Vorstellungen über den Menschen in der Medizin auch deswegen eine Provokation, weil die Medizin über die Zerlegung der innermenschlichen Prozesse die Anerkennung als „wissenschaftlich, fortschrittlich und helfend“ erreicht hatte (Hagner 2006: 318). Die scharfe Gegenwehr der Psychiater ist zudem auf die Argumentationsweise der Vertreter psychosomatischer Ansätze zurückzuführen. Dabei wog es gar nicht so schwer, dass sich Weizsäcker und Mitscherlich auf die klinische Erfahrung beriefen; dies war im Bereich der Nervenheilkunde keineswegs ungewöhnlich. Doch waren viele ihrer Aussagen für die Referenten aus der Psychiatrie kryptisch formulierte, schwer nachprüfbare Behauptungen geblieben. Insbesondere mangelte es an konkreten Belegen über die angenommenen Wirkungszusammenhänge. Die in den Vorträgen herangezogenen Krankengeschichten hatten lediglich der Illustration von Heilungserfolgen gedient; in ihnen fehlten präzise Aussagen zu den angewendeten Verfahren. Mitscherlich hatte beispielsweise in seinem Vortrag zwar „biographisch genaue Anamnesen“ gefordert und von einer schon zitierten „geduldig methodisch exakt geführten psychoanalytischen Behandlung“ gesprochen, dann aber die für die Evidenzbezeugung wichtigen Punkte nicht näher ausgeführt. Auch blieb unklar, was er mit „experimentellen Erfahrungen“ überhaupt meinte (Mitscherlich 1949: 29).

Mitscherlich sah sich aber nicht nur mit der grundsätzlichen Skepsis der Psychiater konfrontiert, sondern hatte sich auch mit dem feingliedrig ausgearbeiteten Vorschlag des Internisten Paul Martini auseinanderzusetzen. Dessen Methodik der therapeutisch-klinischen Forschung beinhaltete Forderungen nach der Wahl zuverlässiger Kriterien, nach eindeutigen Versuchsanordnungen, methodologischer Stringenz sowie nach der Befolgung der Gesetze der (kausalen) Logik und der Erkenntnistheorie.^16^ Martini wandte sich gleich zu Beginn seines Vortrags direkt an Mitscherlich – wohl auch, weil er von diesem zuvor ebenfalls persönlich adressiert und herausgefordert worden war. Mitscherlich hatte ihm ein lediglich „affektdurchdrungenes“ Urteil über die Psychotherapie vorgeworfen (Mitscherlich 1949: 33). Martini ging jedoch zunächst auf Mitscherlich und Weizsäcker zu, indem er die Relevanz psychosomatischer Einflüsse anerkannte. Nicht deren Existenz, sondern die Reichweite der psychosomatischen Aussagen sei ungewiss und ihre Methode fragwürdig, da die angewandten Beweisführungen nicht „ausreichend und richtig“ seien. Auch wenn der Nachweis von „Regelhaftigkeiten“ in diesem Bereich anhand von „ganz individuellen Untersuchungen“ stets schwierig zu erbringen war, forderte Martini dennoch ein, sich um diese zumindest zu bemühen. Es sei nicht hinzunehmen, dass die Vertreter der Psychosomatischen Medizin den deutschen Psychotherapeuten darin folgten, sich „in besonders radikaler Weise“ gegen die Kausalität zu positionieren und eine bedenkliche „Bereitschaft zur kausalen Verknüpfung vorhergehender, gleichzeitiger oder auch einander nachfolgender Phänomene“ zu zeigen. Sie hatten, so Martinis Vorwurf, das „Streben nach Evidenz“ weitgehend aufgegeben und verzichteten großzügig auf „den Beweis der Allgemeingültigkeit“. Martini insistierte demgegenüber auf der Reproduzierbarkeit wissenschaftlicher Ergebnisse, auf der Kausalität in den theoretischen Begründungen und auf ein vergleichend-systematisches und kontrolliertes Vorgehen. Er forderte eine Psychotherapie auf dem gesicherten Boden der Kausalität, eindeutige Versuchsanordnungen und psychoanalytische Vergleichsstudien an „Gesunden“. Nur auf einer solchen Basis ließe sich entscheiden, „in welchem Umfang seelische Alterationen überhaupt mit Krankheiten, sei es neurotischer oder somatischer Art, nicht nur zusammen fallen können, sondern wahrscheinlich zusammen fallen“. Einen eigenen Beurteilungsmaßstab für die Erforschung psychosomatischer Vorgänge lehnte er hingegen ab. Die klinische Medizin beruhte für ihn nicht bloß auf einem Vorurteil, war nicht lediglich „eine Sache der Gewohnheit“ oder „eine Frage des Denkstils“. Daher könne man ihr auch nicht einfach das Recht absprechen, „die Erfolge und die Heilmethoden der Psychotherapie zu zensurieren“. Die Gesetze der Logik und der Erkenntnistheorie seien für alle verbindlich (Martini 1949: 51–55).

Mitscherlich erhob nun wiederum die Stimme gegen diese, wie ihm schien, unpassenden Forderungen und Vorwürfe. Die von Martini formulierten Mindeststandards ergaben nur auf Basis eines Krankheitsverständnisses Sinn, das – wie in der klassischen Schulmedizin – davon ausging, Krankheiten seien stets das Ergebnis einer im Körper beginnenden Leistungsveränderung. Mitscherlich sprach daher sogar von zwei nichtkompatiblen „Wissensformen“: einer „konservativen“ und einer „neuen“ (Aussprache 1949: 81). Er bezweifelte zudem den Nutzen statistischer Verfahren zur Evidenzbezeugung. Eine einfache Kausalität, das hatte schon Weizsäcker betont, war in der Darstellung psychologischer Verhältnisse und Prozesse nicht zu erreichen (Weizsäcker 1949: 20). Eine überpersonale Kausalität, so Mitscherlich, gebe es im Krankheitsgeschehen ebenfalls nicht, da nicht jeder Körper in gleicher Weise auf nichtintegrierte Gefühls- und Triebregungen reagiere (Mitscherlich 1949: 29 f.). Zudem versage die Kausalanalyse dabei, die Frage nach dem individuellen Krankheitssinn – und das stand für Mitscherlich ja im Zentrum von Forschung und Therapie – zu beantworten. Mit kausalem Denken allein könne man der Einmaligkeit des (erkrankten) Subjekts nicht gerecht werden. Auch stellte die Statistik – der Martini eine große Bedeutung bei der Evidenzbezeugung beimaß – für Mitscherlich „noch keine ausreichende Kontrolle der Wissenschaftlichkeit“ dar; sie schien ihm leicht manipulierbar und nur begrenzt aussagekräftig. Gerade an ihr könne man erkennen, dass es beim Einsatz von Instrumenten „ganz außerordentlich auf den an[käme], der sie handhabt“. In Statistiken nämlich durchmische sich „die angebliche Objektivität unbemerkt mit subjektiven Momenten“ (Aussprache 1949: 81).

Mitscherlich bezweifelte aber keineswegs, dass es sinnvoll sein könne, Theorien durch empirische Erhebungen und die mathematische Auswertung gewonnener Daten zu bestätigen beziehungsweise zu falsifizieren. Auch hielt er es nicht für angebracht, die strenge Kausalitätsanalyse gänzlich aus der psychosomatischen Krankheitsbetrachtung zu verbannen. Seine Aussage, die statistische Methode sei für die Psychotherapie „nur sehr modifiziert anwendbar“, wollte er daher nicht so interpretiert sehen, als ob die Forschungsaussagen in der Psychosomatischen Medizin nicht zu kontrollieren und zu gruppieren wären. Das, so Mitscherlich, geschehe bereits größtenteils: Wie in „jeder Wissenschaft“ könne auch im Bereich der Psychotherapie eine Erfahrung nur dann Gültigkeit beanspruchen, „wenn sie kontrollierbar ist“. Den Nachweis von „Regelhaftigkeiten […] aus der Übersicht über viele ähnliche Erlebnisse“, wie sie Martini gefordert hatte (Martini 1949: 51), verschob er indes auf später. Auch fehlte in Mitscherlichs Aussagen Genaueres zu den beabsichtigten Modifikationen der statistischen Methode und er blieb Spezifikationen darüber schuldig, wie eine intersubjektiv nachprüfbare Kontrolle gewährleistet werden sollte. Er verwies lediglich darauf, dass man dieses Problem kaum abstrakt erörtern könne, sein Vortrag dafür also nicht der richtige Ort wäre. Man habe die Erfahrungen in den Kliniken abzuwarten. Erst dann werde „das Problem der Wissenschaftskontrolle“ gestellt und diskutiert werden können (Aussprache 1949: 81).^17^

Mitscherlich schränkte seine Aussagen dann auch sogleich ein: Menschenbild und Methodik der Medizin dürften nicht zulasten des agierenden, Wirklichkeit kreierenden Subjekts gehen und zu einer Ausblendung des Lebensschicksals des einzelnen Patienten führen. Da sich der „Mensch als Subjekt“ auch in der Krankheit verwirkliche, sei der Erkrankte nicht lediglich „der fremden Objektivität materieller Geschehnisse unterworfen“. Würde man Martinis Anforderungen an die Wissenschaftlichkeit auf die „Psychologie des Unbewußten“ übertragen, wäre dies deren Ende. Symbole ließen sich nicht objektivieren, ja seien von sich aus so vieldeutig, dass allein der Patient über ihren Sinn und ihre Interpretation entscheiden könne. Ein Analytiker, der mit Symbolen wie mit festen Bedeutungsnormen umgehe und seine vorgefertigten Interpretationen dem Patienten aufnötige, sei „ein Stümper“ (Aussprache 1949: 77–81).

## Anhaltende Kritik und enttäuschte Hoffnungen

Nach dem DGIM-Kongress im Jahr 1949 glaubten die Vertreter der Psychosomatischen Medizin, einen Durchbruch erreicht zu haben. Für Weizsäcker hatte sich auf dem Wiesbadener Internistenkongress „ein neuer Zustand der Medizin“ offenbart. Seiner Meinung nach konnte man Krankheiten künftig nicht mehr ausschließlich naturwissenschaftlich erklären und behandeln. Sie würden „beim Menschen eine Weise seines Menschseins“ darstellen, „an der er mit allen seinen Teilen, Leib und Seele, Körper und Geist, bewußt und unbewußt beteiligt ist“ (Weizsäcker [1950]).^18^

Doch die Kritik am Wissenschaftsverständnis der Psychosomatischen Medizin riss nicht ab. Der Kreis der standespolitisch einflussreichen Psychiater wies Erklärungsmodus und Reformanspruch der Psychotherapeuten und Psychosomatiker weiterhin von sich. Sie zweifelten an der Glaubwürdigkeit der aus den Fallanalysen abgeleiteten Ergebnisse, gestanden ihnen lediglich eine gewisse anekdotische Evidenz zu. Nicht nur die vorgebrachten Argumente sind hierbei relevant. Auch der unversöhnliche und nicht selten persönlich verletzende Ton der Debatte fällt ins Gewicht. Offenkundig verhärteten sich in den polemischen Scharmützeln die Fronten zwischen universitärer und praktischer Psychiatrie auf der einen und den Verfechtern tiefenpsychologischer Verfahren auf der anderen Seite. So entwickelten sich die sich verstärkt als Psychopharmakologie verstehende Psychiatrie einerseits und die Psychosomatik/Psychotherapie andererseits auch institutionell voneinander getrennt (Roelcke 2020: 53).

Nicht nur das Gros der Psychiater verhielt sich geradezu feindlich gegenüber der tiefenpsychologischen Psychotherapie und der Psychosomatischen Medizin. Umgekehrt tendierten auch deren Vertreter zur Selbstabschottung gegenüber der Psychiatrie. Wie man an Alexander Mitscherlich gut sehen kann, reagierten sie nicht bloß auf Angriffe. Einige Kritiker, auch aus den Reihen der Psychiatrie, lobten Psychosomatik und Psychotherapie dafür, eine neuartige Vielfalt der Erscheinungsformen von Krankheit sichtbar gemacht zu haben (z. B. Wendt 1952: 351). Nicht diese waren umstritten, sondern die verwendeten hermeneutischen Verfahren zur retrospektiven Deutung krankhafter psychischer und somatischer Phänomene. Und noch ein weiterer Punkt ist relevant: Auch die Psychiater konnten im eigenen Tätigkeitsfeld oftmals weder die exakten Ursache-Wirkungsbeziehungen bei der Genese psychiatrischer Krankheitsbilder eruieren, noch eine klare Indikationsstellung oder eine kausale Therapie gewährleisten. Entscheidend für das Verständnis der nachfolgenden Ausführungen ist aber vor allem, dass so manche Hoffnung von 1949 schon wenige Jahre später der Enttäuschung wich: „ärztliches Handeln aus psychosomatischem Wissen“ war, wie de Boor 1958 schon fast resigniert und die tatsächlichen Erfolge in der Institutionalisierung der Psychosomatik ausblendend festhielt, „randständig“ geblieben. Auch wenn man die Erfahrungen in diesem Bereich vertieft und die „zweifellos noch fragmentarischen Erkenntnisse gemehrt“ habe, so werde den deutschen Psychosomatikern fortwährend vorgeworfen, sie betrieben „eine mystische Naturphilosophie“ (de Boor 1958: 511 f.).^19^

## Verfeinerte Kritik am „organmedizinischen Denkmodell“

Schon in seinen Stellungnahmen aus der zweiten Hälfte der 1940er Jahre ging Mitscherlich davon aus, dass neue epistemische Prämissen und ein grundlegend anderes Krankheitsverständnis unabdingbar waren, wolle man die Entstehung von Krankheiten in einem Korrelationsfeld von Erlebnis und diesem Erlebnis zugeordneten körperlichen Leistungen verstehen. Auch nachfolgend betonte er, dass, versuche man seelische und leibliche Erregungen als „Aspekte *eines* Vorgangs“ zu begreifen, man sich plötzlich einem Facettenreichtum gegenübersehe, den man zwar beschreiben könne, dessen Wirkungsmechanismen aber einem simplen Verständnis von Kausalität zuwiderliefen. Wenn psychosomatische Erkrankungen dadurch entstünden, dass eine psychische Erregungskomponente die individuelle Spannweite physiologischer Variationen überstieg und so eine pathologische Reaktion provozierte, benötige man sowohl ein verändertes Konzept der Pathogenese als auch eine größere Offenheit für die vielfältigen Bedingungszusammenhänge. Wolle man den Menschen und die in seinem „Schicksalsvollzug“ von ihm entwickelten Störungen und Pathologien in ihrer ganzen Komplexität verstehen und therapieren, war Mitscherlich zufolge eine völlig „neue Wissenschaftsform“ mit neuen Vorstellungen über das Zusammenwirken simultaner Prozesse und einer anderen als der bisherigen Wissenschaftssprache vonnöten. Ferner forderte er einen Wechsel des klinischen Beobachterstandorts, neue ärztliche Denkgewohnheiten und eine modifizierte Haltung als Forscher und Therapeut (Mitscherlich 1966/1969b: 56 f.). Wenn damit auch in den Auseinandersetzungen um die Kriterien für valides Wissen vor allem Argumente wieder aufgegriffen wurden, die ohnehin bereits zuvor formuliert worden waren, so änderten sich doch deren Wertigkeiten in der Gesamtargumentation deutlich. Unter Hinzuziehung neuer Erkenntnisse aktualisierte Mitscherlich seine Kritik nicht nur, sondern plausibilisierte sie auf andere Art und Weise. Das zeigt sich zunächst in der zweiten Hälfte der 1950er Jahre, als er sich noch einmal dezidiert mit Martinis Forderungen nach einer an die Naturwissenschaften angelehnte Methodik in der medizinischen empirischen Forschung auseinandersetzte.

### „Unwissentliche Versuchsanordnung“ und „unwissentliche Kontrolle“

Unmittelbarer Anlass für die neuerliche Konfrontation mit Paul Martini war dessen Rede zur Eröffnung der Karlsruher Therapiewoche 1957. Hier wiederholte Martini die Voraussetzungen für eine „rationale Therapie“, und Mitscherlich reagierte darauf. Doch seine Situation war nun eine gänzlich andere. Er hatte der Psychoanalyse in der Bundesrepublik zu Ansehen verholfen und zugleich selbst erheblich von der Etablierung einer eigenständigen psychosomatischen Disziplin profitiert. Die inhaltliche Schnittmenge zur medizinischen Anthropologie Weizsäckers hatte weiter abgenommen. Er war nun ein überzeugter Freudianer, der 1958 eine Lehranalyse begann.^20^ Zudem war er als öffentlicher Intellektueller etabliert und erreichte mit der jetzt mit *Medizin ohne Menschlichkeit *betitelten Neuauflage der Dokumentation zum Nürnberger Ärzteprozess im Speziellen und seiner Kritik an der Schulmedizin und ihren Modellen im Allgemeinen viel weitere Kreise als bisher. Dadurch nahmen seine Möglichkeiten, Bündnispartner außerhalb der Medizin zu rekrutieren, deutlich zu.

Vieles an Mitscherlichs nun getätigten Aussagen war bereits bekannt: Mit den Therapie- und Forschungsstandards Martinis seien stets nur Angaben über einen begrenzten Ausschnitt der Wirklichkeit möglich. Diese seien zwar jede für sich in ihrem jeweiligen Geltungsbereich korrekt und experimentell immer wieder überprüfbar, doch wäre die Summe der separaten Ursache-Wirkungszusammenhänge noch lange nicht gleichbedeutend mit der „Lebensganzheit“ der Patienten. Mit ihren kleinteiligen Untersuchungen sei die „Schulmedizin“ zwar zu Aussagen neuer Qualität gelangt, ihr reduktionistisches Vorgehen sei aber auf Kosten des individuellen Kranken gegangen. Die medizinische Wissenschaft, so Mitscherlichs Vorwurf, habe die Produktionsbedingungen ihres Wissens vergessen. Sie erfasse nur scheinbar objektiv die vorhandene reale Welt und werde der Komplexität ihres Untersuchungsgegenstands nicht gerecht. Wie auch schon zuvor bestritt er nicht den möglichen Nutzen kausaler Analysen. Auch wandte er sich nicht generell gegen Martinis „wissenschaftlichen Purismus“. Er hielt den Wunsch nach methodischer Prüfung und das Ideal einer „so stark wie möglich gesicherten Heilmethode“ für berechtigt und lobte Martinis Ansatz als den Ausdruck eines „Ordnungsstreben[s] von hoher geistiger Qualität“ (Mitscherlich 1958/1983d: 116, 118, 120).

Mitscherlich begnügte sich aber nicht mit der Wiederholung bereits zuvor geäußerter Argumente. Deutlicher als im Jahr 1949 setzte er sich jetzt konkret mit den von Martini geforderten Studienbedingungen auseinander. In seiner Erwiderung lehnte er dessen Forderung nach einer „unwissentlichen Versuchsordnung“ im eigenen Bereich therapeutischen Handelns ab. In diesem seien erstens Vorbeobachtungs- und Vergleichszeit nicht eindeutig voneinander zu trennen, könnten zweitens nicht sämtliche Rahmenbedingungen stabil gehalten, noch drittens homogene Untersuchungs- und Vergleichsgruppen gebildet werden. Was in der somatischen Therapie oder der Pharmakotherapie leicht zu gewährleisten sei, ließe sich bei psychotherapeutischen Experimenten von vornherein nicht einhalten. Man dürfe daher nicht einfach, wie Martini, „ein Handlungsmodell von einer Grundkonzeption auf eine andere […] übertragen“.^21^ Mitscherlich stellte nun seinerseits Forderungen an die klinische Medizin. Sie habe „selbstkritisch Methoden [zu] entwickeln und [zu] lehren, die den Arzt anweisen, wie er mit dem Kranken umgehen muß, um von ihm inhaltlich die Evidenz zu bekommen, daß er ein Mitmensch ist“ (Mitscherlich 1958: 729). Die „Organmedizin“ habe „eine unwissentliche Kontrolle“ einzuführen. Schließlich habe jede „rationale Therapie“, so Mitscherlich, den Arzt selbst mit in die Untersuchung einzubeziehen. Nur dann könne sie überhaupt Aussagen darüber treffen, was in der Beziehung zwischen Arzt und Patient „therapeutisch wirkt, was seine Wirkung verfehlt oder gar schadet“ (Mitscherlich 1958/1983d: 122).^22^*Participant observers* – etwa in der Feldforschung erfahrene Sozialpsychologen –, die ohne Kenntnis der Ärzte als Patienten das Stationsgeschehen und den Arzt-Patienten-Kontakt beobachten, sollten mit ihren Analysen zeigen, was „tatsächlich“ und nicht nur „dem Willen der Versuchsleiter nach auf einer Station passiert“ (Mitscherlich 1958/1983d: 130).^23^ Mitscherlich versuchte hier seinerseits einen allgemeinen Standard zu setzen. Er hielt damit den Vertretern der „Organmedizin“ einen Spiegel vor. Augenzwinkernd verwies er darauf, dass auch Martini gepredigt habe: „Wehe dem Wissenschaftler, wenn er […] seine Simplifizierung überhaupt nicht bemerkt“ (ebd.).

### „Purism of Method“

Kurz darauf, im April 1959, hielt Alexander Mitscherlich auf dem von Arthur Jores geleiteten Europäischen Symposium für psychosomatische Forschung einen Vortrag über Forschungsmethoden in der Psychosomatik. Da psychoanalytische Experimente grundsätzlich unmöglich seien, so betonte der nach Profilierung vor einem internationalen Publikum trachtende Mitscherlich gleich zu Beginn, würden sich psychoanalytische Theorien stets nur auf die klinische Beobachtung stützen können. Zu dieser Zeit war eine solche Stellungnahme eine klare Gegenposition zur US-amerikanischen Psychosomatik, die Mitscherlich zufolge zu experimentell, zu statistiklastig und zu stark an der Organmedizin orientiert sei. Das verwundert auf den ersten Blick, denn erstens hatte Mitscherlich zuvor die Heidelberger Klinik nur durch die finanzielle Unterstützung der US-amerikanischen Rockefeller-Foundation aufbauen können. Zweitens war er selbst stark von der in den USA florierenden Psychoanalyse und dabei insbesondere durch das Werk Franz Alexanders (1891–1964) beeinflusst worden. Mitscherlich hatte sich und seinen Mitarbeitern zudem, drittens, auch immer wieder Reisen in die USA gegönnt und sich für Lehranalysen und Auslandsaufenthalte in den Vereinigten Staaten ausgesprochen. Außerdem nutzte er „jede sich bietende Möglichkeit“, um ausländische Analytiker für Vorträge, Forschungs- und Ausbildungsaktivitäten in die Bundesrepublik einzuladen (Hoyer 2008: 279). Schließlich hatte er selbst mehrmals mit dem Gedanken gespielt, in die USA überzusiedeln. Die Angebote von dort hatte er zwar immer wieder ausgeschlagen und lediglich dazu genutzt, seine Position im eigenen Land zu festigen, doch hatte er den Austausch mit dem angloamerikanischen Raum forciert und Anschluss an die dortigen Forschungsfragen und Theoriebildungen gesucht. Nun warf er den Forschern aus den USA jedoch vor, sie hätten zu große Angst davor, am zentralen Tabu zu rütteln. Sie, die ja frühzeitig von einer kliniknahen Institutionalisierung profitiert hatten, würden sich nicht trauen, die kollektiv geteilten Ansichten über Wissenschaftlichkeit direkt anzugreifen.

Das war aber nur ein Nebenschauplatz, auf dem Mitscherlich sich vor seinen internationalen Zuhörern rasch verorten konnte und auf dem er sich nicht lange aufhielt. Zentral war für ihn indes weiterhin die Abgrenzung von der Beobachtungsweise der „konventionellen Medizin“. Die psychoanalytische und die *conventional medicine* würden zwar das Objekt (den Menschen) und das Ziel (ihn zu heilen) teilen, seien aber ganz unterschiedlicher Ansicht darüber, wie über das Objekt Aussagen zu formulieren und wie eine Therapie zu konzipieren sei. Mitscherlich wehrte sich mit scharfen Formulierungen gegen die Zusammenarbeit von Organmedizinern und Psychoanalytikern. Eine solche Kooperation biete den Patienten keinen Nutzen, für die Psychoanalytiker sei sie gar voller Gefahren. Daher wandte er sich auch gegen das „scientific Esperanto, using the grammar of experimental research and statistics“ und gegen den „premature methodological eclecticism“, den er der gegenwärtigen Psychoanalyse attestierte. Stattdessen plädierte er für getrennte Wissenschaftssprachen und einen „purism of method“. Man möge, so seine Empfehlung, die Kraft nicht damit verschwenden, die Symptomentstehung und das Verschwinden der Symptome zu erklären. Das verstärke zwar möglicherweise die Isolation der Psychoanalytiker, doch sei aus einer solchen Position schon oft Großes erwachsen: Auch die Schöpfer der modernen Malerei, so Mitscherlichs bezeichnende Analogie, hätten sich bewusst außerhalb des Kanons verortet und sich so von den etablierten Künstlern den Vorwurf eingehandelt, sie könnten nicht zeichnen (Mitscherlich 1960: 21–36, 40 f.).

Nach dieser Vorrede umriss er, welche Konsequenzen aus den bisherigen empirischen Erfahrungen für die psychoanalytische Theoriebildung zu ziehen seien. Da er anders als in der unmittelbaren Nachkriegszeit nun sicher war, dass es keine typischen Krankheitscharaktere gebe und dass das Symptom selbst auch keine Auskunft über den auslösenden unbewussten Konflikt erteile, gab es für ihn nun überhaupt keinen Grund mehr, das klinische Klassifikationssystem in das psychoanalytische Schemata zu integrieren. Die Erfahrung zeige, dass zwar relativ ähnliche Konflikte zu relativ ähnlichen Symptomformationen führten, die Symptome stünden aber mitunter für ganz unterschiedliche Verarbeitungsformen des Konflikts. Daher müsse die gesamte Persönlichkeitsstruktur in Diagnose und Behandlung einbezogen und auch in der Forschung berücksichtigt werden. Heilung, so Mitscherlich weiter, dürfe auch nicht mit dem Verschwinden von Symptomen verwechselt werden. Eine erfolgversprechende Therapie habe die individuellen adaptiven Fehlleistungen soweit wie möglich mitzubehandeln. Jede Diagnose habe genau Aufschluss über die Pathogenese zu geben und jede Therapie habe das Symptom auf den verursachenden Konflikt zurückzuführen (Mitscherlich 1960: 37–39). Erst die Heilung verifiziere damit die Deutung: „The […] only possible criterion of the *correctness* of an interpretation of the psycho-dynamics of a psychosomatic symptom is a cure.“ (Mitscherlich 1960: 40) Hinter vielen wolkigen Worte blieb indes auch in diesem Vortrag unklar, was „Heilung“ konkret bedeutet und nach welchen Kriterien sie verifiziert werden kann.

### Die konventionelle Medizin als „Majoritätsreligion“ und die naturwissenschaftliche Methode als Abwehrmechanismus

Schon auf der DGIM-Tagung im Jahr 1949 hatte sich Mitscherlich an „alle Kritiker aus dem Lager der Psychiatrie“ gewandt und die Fundamente der naturwissenschaftlichen Medizin als „höchst willkürliche Konvention […] im Denken“ bezeichnet, die auf einer „zweifelhafte[n] Arbeitsteilung zwischen den Heilbemühungen um den Menschen“ beruhe (Aussprache 1949: 79). Diese Fährte nahm er in den 1960er Jahren wieder auf. Jetzt kreiste Mitscherlichs Kritik um andere Fragen, bezog neue Erklärungsmodelle mit ein und gehorchte einer neuen Logik und Dramaturgie. Angesichts der weithin wirkungslosen Interventionen der Vorjahre richtete sich sein Erkenntnisinteresse auf den Tatbestand, dass die Mediziner trotz aller – nicht zuletzt von ihm selbst aufgezeigten – Probleme und Ungereimtheiten an den Denkkonventionen ihres Fachs festhielten. Wenn, wie er annahm, die Schulmedizin als Wissenschaft an einem Punkt angelangt war, an dem sie entweder an ihren inneren Widersprüchen zerschellen oder sich neuen Modellen, Begriffen und Denkgewohnheiten öffnen werde, bedurfte es einer Erklärung, warum die Mediziner trotz aller offenkundigen Krisensymptome noch immer an bisherigen Modellvorstellungen festhielten, statt sich der Psychosomatik zuzuwenden. Nicht mehr die Zumutungen^24^ und Ansprüche der Psychosomatischen Medizin oder „Probleme des Wissens“ standen daher im Fokus, sondern die Frage, warum der Denkstil der „Organmedizin“ so erstaunlich stabil blieb (Mitscherlich 1966/1969c: 9).

Den Weg zu einer Antwort ebneten sozialpsychologische und medizinsoziologische Ansätze.^25^ Auf sie rekurrierend ging Mitscherlich davon aus, dass medizinisches Wissen von einer eindeutig bestimmbaren gesellschaftlichen Gruppe generiert, universalisiert und weitervermittelt werde. Auch die Gründe für die ablehnende Haltung gegenüber der Psychosomatischen Medizin waren dann in der besonderen sozialen Stellung der ärztlichen Berufsgruppe und in deren spezifischen Sozialisationswegen zu suchen. Unter Bezugnahme auf Helmut Schelsky, René König, Robert K. Merton und Michael Balint (Merton et al. 1956; Balint 1957; Schelsky 1958; König 1958) betonte er, dass eine gefestigte Berufsidentität stets auf der Übernahme von Vorurteilen beruhe und damit einer „Indoktrinierung“ gleichkäme. Nur wenn der Forscher und Arzt die „dogmatisierte Prämisse“, dass Krankheiten stets Körperkrankheiten seien, anerkenne, könnten seine Aussagen und Handlungen überhaupt darauf hoffen, den „Charakter wissenschaftlicher Legalität“ zugesprochen zu bekommen (Mitscherlich 1966/1969b: 54). Da der „Grad der Widerstandslosigkeit“ gegen diese Prämisse über Karrieren im Wissenschaftsbetrieb und an den Kliniken entscheide, werde die reibungslose Einpassung in das Bestehende und nicht der konstruktive Widerspruch belohnt (Mitscherlich 1966/1969a: 48).

Mitscherlichs sozialpsychologisch und medizinsoziologisch erweiterte Kritik war intellektuell anregend, resultierte aber nicht aus eigenen empirischen Untersuchungen.^26^ Sie speiste sich nicht aus der konkreten Beobachtung der Wissensproduktion oder einer Analyse der wissenschaftlichen Kommunikation zur Stabilisierung wissenschaftlicher Fakten. Weil er keine Mikrosoziologie des akademischen Arbeitens und Lehrens betrieb, formte sich in seiner Analyse eine homogene Gruppe an Wissensträgern, die nicht nach Generationszugehörigkeit, Geschlecht oder Herkunft differenziert werden musste und deren Mitglieder alle den gleichen Interessen folgten. Auf Basis dieser Annahme war es folgerichtig, davon auszugehen, dass die berufsgruppenspezifischen Konventionen in der Medizin zu Dogmen erstarrt seien und das Fach im Stillstand enden werde. Das hatte er schon in seiner Schrift über Freiheit und Unfreiheit in der Krankheit behauptet (Mitscherlich 1946/1977: 32, 34), doch nun baute er sein Argument erheblich aus. Ähnlich wie in einer „Majoritätsreligion“, so Mitscherlich über die „konventionelle Medizin“, reagierten deren Anhänger auf Kritik mit emotionaler Abwehr, würden notwendige Elemente der Weiterentwicklung als feindliche Gesinnung interpretieren und Neuerer zu Ketzern abstempeln.^27^ Mitscherlich zeigte sich in solchen Passagen nicht gerade als gelassener Beobachter. Die Auseinandersetzung mit seinen Gegnern erfolgte, wie so oft, im Ton einer Abrechnung. Die Rollen waren dabei klar verteilt: Mitscherlich selbst war der missverstandene „Neuerer“, seine Opponenten waren Repräsentanten einer unbeweglichen Berufsgruppe, die damit beschäftigt waren, ihren Status und die Richtigkeit ihrer Denkmodelle durch einen einheitlichen, sozial und habituell geschlossenen Bildungsweg abzusichern und die an den Universitäten in „konservativer Selbstfesselung“ eine „wohlorganisierte ‚geschlossene Gesellschaft‘“ formten (Mitscherlich 1966/1969a: 37; Mitscherlich 1966/1969b: 55).

Mitscherlich interessierte sich nun auch dafür, wie die seiner Ansicht nach immer stärker werdenden Abwehrreflexe aus der Riege der etablierten Wissenschaftler und Kliniker erklärt werden konnten. Mittlerweile ganz Psychoanalytiker, interpretierte er die „naturwissenschaftliche“/„rationale“ Methode nicht mehr allein als Wissens- oder Glaubenssystem, sondern als Mechanismus, um das „beängstigend Unbekannte ab[zu]wehren und alles Licht auf einen, eben den mit Erfolg beschriebenen und manipulierbar gewordenen Teilausschnitt der Welt fallen [zu] lassen“. Den Vertretern des hegemonialen Modells gelänge es so, sich Rationalität zuzuschreiben, das eigene „Selbstgefühl“ zu steigern und Angst abzuwehren. Der zutiefst verunsicherte Arzt zeige eine „Tendenz, durch Besiegung der Krankheit seinen Narzißmus zu stärken und auch auf diese Weise den Angstdruck zu lindern“. So verkäme die wissenschaftliche und therapeutische Tätigkeit in der „Organmedizin“ immer mehr zu einer „voyeuristische[n] Stimulierung und Befriedigung“. Da in der aktuellen Krise des bisherigen Wirklichkeitsmodells vermehrt Enttäuschungen drohten, seien immer heftigere Formen der Abwehr – „Rationalisierung“ und „Verleugnung der Realität“ – zu beobachten und zu erwarten (Mitscherlich 1958/1983c: 119 f.; Mitscherlich 1966/1969b: 66).

Die „große […] Haltbarkeit“ der „Vorurteile“, die in der Medizin mit der „Vorstellung von Wissenschaftlichkeit“ verknüpft waren, seien so entweder – in sozialpsychologischer Sicht – Ergebnis von Machtsicherungsstrategien oder – psychoanalytisch betrachtet – Effekte eines hochwirksamen „psychische[n] Abwehrsystem[s]“, das gegen die „harte Kränkung“ der psychosomatischen und psychoanalytischen Betrachtungsweise anspringe. Auf Basis dieser Urteile kam Mitscherlich zu einem lesenswerten Plädoyer für mehr Uneinheitlichkeit und Ambiguitätstoleranz (Mitscherlich 1966/1969b: 59 f., 63).^28^ Zugleich musste er sich dank dieser Deutungen aber auch nicht mehr mit einer Reihe inhaltlich-methodischer Einwände beschäftigen.^29^ Martinis Insistieren auf einer gesicherten Wissensbasis der medizinischen Forschung und Therapie war demnach – ohne dass Mitscherlich dies aussprechen musste – bloß noch ein letztes Rückzugsgefecht jener „konservativen Wissensform“, die Mitscherlich schon 1949 als überholt und obsolet bezeichnet hatte.

Eine letzte epistemologische Wendung folgte: Mitscherlich kritisierte später die tiefenpsychologischen und biografischen (nicht aber psychoanalytischen!) Ansätze dafür, dass sie nicht im Labor überprüft werden könnten und erst in der Rückschau plausibel wurden. Offenbar verschob sich, als in der psychologischen Forschung standardisierte Testverfahren immer üblicher wurden, auch die Bewertung von Verfahren, die auf biografischer Einmaligkeit insistierten und dem Trend zu standardisierten Verfahren widersprachen. Im Mittelpunkt auch dieses Konflikts standen divergierende Ansichten über den wissenschaftlichen Standard zur Evidenzherstellung und die Grenzen der Psychologisierung des Somatischen. Mitscherlich erkannte nun an, dass sich auch die psychosomatische Forschung an den gültigen Standards zur Überprüfung von Verfahren und Ergebnissen messen lassen musste, wollte sie das Ziel der wissenschaftlichen und nicht nur der öffentlichen Anerkennung nicht aus den Augen verlieren. Damit revidierte er zugleich den vormaligen Anspruch der Psychosomatik. Statt die gesamte Medizin zu revolutionieren, beschied er sich schließlich mit der Etablierung eines eigenständigen Fachs (Hitzer 2020: 65–67).

## Fazit

In den Auseinandersetzungen um die Psychosomatische Medizin ging es um Menschenbilder, um die Relevanz von Perspektiven, Methoden und Wissen sowie um die Validität von Behauptungen und Begründungen. Die Psychosomatische Medizin trat dabei in der unmittelbaren Nachkriegszeit mit einem großen Versprechen auf: Sie beabsichtigte, *der* Perspektivenwechsel in der Betrachtung menschlicher Erkrankungen zu sein. Mehrheitlich waren ihre Vertreter daher auch davon überzeugt, dass die psychosomatischen, tiefenpsychologischen und psychoanalytischen Verfahren nicht dadurch vom medizinischen Establishment anerkannt werden würden, dass sie sich an deren Modelle anpassten. Stattdessen stellten sie immer wieder den gesellschaftlichen und epistemischen Sonderstatus des „organmedizinischen Wissens“ infrage.

Die damit verbundene Grundsatzkritik am naturwissenschaftlich orientierten Menschenbild und an der naturwissenschaftlichen Erkenntnisweise war keineswegs unbeweglich. Das zeigt sich an Alexander Mitscherlichs Stellungnahmen besonders deutlich. Seine hier untersuchten Auseinandersetzungen mit Paul Martini belegen,^30^ dass sich die epistemologischen Positionen über zwei Jahrzehnte hinweg verfeinerten und verschoben. Indem Mitscherlich seine Argumentation an die jeweils aktuelle gesellschaftliche, wissenschaftliche und politische Konstellation anpasste, machte er sie immer wieder auf neue Weise anschlussfähig. Dass sich dadurch auch Mitscherlichs theoretisch-methodische Referenzpunkte änderten, brachte ihm seitens seiner Biografen den Vorwurf ein, seine wissenschafts- und erkenntnistheoretischen Argumentationen seien „unausgegoren“ gewesen. Auch wurde die bei ihm unübersehbare enge „Verknüpfung von medizinischen Phänomenen mit moralischen Kategorien“, der „weltanschauliche Überschuss“ sowie die „metaphorische Aufladung seiner Texte“ kritisiert (Hoyer 2008: 183; Dehli 2007: 102, 104). Doch um sie zu etablieren, verankerte Mitscherlich Psychosomatische Medizin (und Psychoanalyse) bewusst in einem Überschneidungsbereich zwischen Naturwissenschaft, Geisteswissenschaft und Politik.^31^

Die hier zur Analyse von Alexander Mitscherlichs epistemologischen Umorientierungen herangezogenen Auseinandersetzungen bezüglich der Kriterien für relevantes und valides Wissen in der Medizin zeigen, dass es nicht sinnvoll ist, ein mittlerweile hegemoniales Verständnis von Evidenz in der Medizin auf frühere Zeiten zu übertragen und als überzeitlichen Qualitätsmaßstab festzuschreiben. Interessanter und den zeitgenössischen Protagonisten gerechter wird ein Ansatz, der ihre Plausibilisierungsweisen zum Untersuchungsgegenstand macht, statt sie an heutigen – ebenfalls umstrittenen – Standards zu messen. Fragt man offener nach den zeitgenössischen Kriterien für relevantes und valides Wissen, so eröffnet die Nachkriegszeit ein Panorama vielfältiger Ansichten darüber, was eine exakte Beobachtung und was eine schlüssige Beweisführung sei. Gerade die Auseinandersetzungen um die Reichweite der Psychosomatischen Medizin und ihrer Methoden zeigen, dass in den ersten beiden Nachkriegsjahrzehnten keine klar definierte Bedeutung von Evidenz in der Medizin existierte. Es war vielmehr umstritten, welche Äußerungen den Charakter gesicherter wissenschaftlicher Erkenntnis für sich beanspruchen konnten und welche nicht.

## Footnotes

1 Eine Disziplin namens Psychosomatik gab es 1949 noch nicht. Die Bezeichnung wurde zu diesem Zeitpunkt zwar bereits verwendet, setzte sich aber erst Anfang der 1950er Jahre auf breiter Basis durch. Wird der Begriff im Folgenden verwendet, soll er nicht suggerieren, dass die Formen und Grenzen einer Psychosomatischen Medizin bereits erkennbar waren, sondern nur, dass er bereits als Bezeichnung diente, unter der sich unterschiedliche Interpretationen versammeln ließen. Psychosomatische Medizin, analytische Psychotherapie, Tiefenpsychologie und Psychoanalyse wurden von den zeitgenössischen Debattenteilnehmern nicht klar voneinander getrennt. Trotz unterschiedlicher Benennung handelte es sich um ein gemeinsames Handlungsfeld. Die Grenzen waren hier stets fließend, sodass diese Akteure sinnvoll nur zusammen als Gruppe behandelt werden können. Auch von ihren Gegnern wurde der genannte Personenkreis als ein zusammengehöriger wahrgenommen. Der damals übliche Sammelbegriff war Psychotherapie.

2 Dieser Kongress gilt als „Wendepunkt in der Geschichte der psychosomatischen Medizin“, weil sich hier abzeichnete, dass sich die Psychosomatik außerhalb der Psychiatrie und in Nähe zur Inneren Medizin institutionell etablieren würde. Geisthövel & Hitzer (2019: 179).

3 Mit dieser Zweiteilung in eine naturwissenschaftliche und eine anthropologische Psychosomatik setzte er sich auch von dem für ihn verfügbaren neuen anglo-amerikanischen Schriftgut ab – namentlich nannte er Alexander, Dunbar, English und Weiss, Binger und Halliday. Darin, so Weizsäckers Vorwurf, könne man nicht erfahren, „wie der äußere und innere Konflikt – die ja gar nicht trennbar sind – in einer [Krankheit] zum Ausdruck kam, in ihm materialisiert wurde, andere Gedanken und Gefühle dabei entstanden“. Weizsäcker (1949: 16).

4 Weizsäcker spielte mit seiner Formulierung, die Psychosomatik werde „eine tiefenpsychologische sein oder sie wird nicht sein“ auf die berühmten Worte Bernhard Naunyns aus dem Jahr 1905 an: „Die Heilkunde wird eine Wissenschaft sein, oder sie wird nicht sein“. Zitiert nach Hagner (2006: 318).

5 Unter „biographisch“ verstand Mitscherlich, „daß der ‚Charakter‘, die Stimmung, die körperliche Verfassung und Reaktionsweise zusammen gesehen werden“. Mitscherlich (1947: 23).

6 Die Berufung auf den „Sinn“ war für die Vertreter der Psychosomatischen Medizin oftmals lediglich der Hinweis, dass man nicht an ein rein zufälliges und damit sinnloses Krankheitsgeschehen glaube. Mit dem „Sinn“ der Krankheit ging allerdings auch ein Teil der Verantwortung für die Krankheit auf die Betroffenen über. Vgl. Wittmer (1994: 131–133).

7 Dies basierte auf den Annahmen, dass eine „Organkrankheit ein Stellvertreter eines ungelösten Konflikts ist“ und durch eine „gelungene Psychotherapie organischer Krankheit“ auch der Konflikt ans Tageslicht gezogen werde. Dann seien eben auch persönliche und gesellschaftliche Konsequenzen zu erwarten – von der Ehescheidung bis zum „politische[n] Umsturz oder [einer] religiöse[n] Revolution“. Weizsäcker (1949: 21).

8 Mitscherlich benutzte hier sicherlich absichtlich den Begriff der ärztlichen Kunst und nicht den der ärztlichen Wissenschaft. Er begründete aber nicht genauer, warum. Mitscherlich hatte die Psychosomatische Medizin schon zuvor als jene entscheidende Stiländerung in der Krankheitsbetrachtung bezeichnet, durch welche die Ärzte, die bloß noch „Techniker der körperlichen Apparatur“ seien, wieder in die Lage versetzt würden, wirklich zu heilen. Mitscherlich (1946/1977: 92, 94). Auch Weizsäcker ging angesichts der großen weißen Flecken in der psychosomatischen Forschung davon aus, dass sich noch nicht erkennen ließe, wo überhaupt die Grenzen des eigenen Ansatzes lägen. Vgl. Weizsäcker (1949: 20). Der (angebliche) Gegensatz von „Wissenschaft“ und „Kunst“ war schon ein zentraler Topos der Debatten zur „Krise der Medizin“ der 1920er Jahre gewesen. Vgl. Roelcke (2016).

9 Hagner attestiert Viktor von Weizsäcker eine „geradezu verächtliche Abneigung gegen die naturwissenschaftliche Medizin“. Hagner (2006: 335).

10 So konnte die Darstellung des Verhältnisses von Organmedizin und Psychosomatischer Medizin auf wohletablierte Topoi aus der Zeit der Weimarer Republik zurückgreifen. Ein Teil der Argumente findet sich bereits in den Debatten der 1920er Jahre um die „Krise der Medizin“. Seit den frühen 1920er Jahren war über Nutzen und Schaden der naturwissenschaftlichen Betrachtungsweise in der Medizin diskutiert worden. Die Vorwürfe eines einseitigen Fokus auf die Somatik und der Vernachlässigung der „psychologischen und sozialen Dimensionen des Kranksein[s]“ waren wichtige Gemeinplätze dieser Reformbewegung. Es war für die unmittelbare Nachkriegszeit keineswegs ungewöhnlich, nach Orientierung in den scheinbar unbelasteten Traditionen der Medizin der Weimarer Republik zu suchen. Vgl. Roelcke (2020: 52). Zur Vorgeschichte des Ganzheitsgedankens vgl. Harrington (2002). Sie verweist u. a. auf die Kompatibilität ganzheitlicher Konzeptionen mit der völkischen Rhetorik und dem eugenischen Ansatz im Zeichen einer „neuen deutschen Heilkunde“ während des Nationalsozialismus. Zudem konstatiert sie, dass diese Verbindung nach 1945 bewusst verdeckt worden ist. Harrington (2002: 318–355).

11 Mitscherlich begriff die Philosophie als Gegenwissenschaft zum Kausaldenken der Naturwissenschaften. Sein Buch sah er aber selbst vor allem von den Werken Sigmund Freuds inspiriert. Im Vorwort dankte er zudem Curt Oehme für die Vermittlung der „unbestechliche[n] Exaktheit der Naturwissenschaften“. Mitscherlich (1946/1977: 8, 51).

12 Zu den wortgewandtesten und einflussreichsten Kritikern seitens der Universitätspsychiatrie gehörten Ernst Kretschmer (1888–1964), Ernst Speer (1889–1964), Kurt Kolle (1898–1975), Jürg Zutt (1893–1980) und Kurt Schneider (1887–1967). Zur Vorgeschichte und Einordnung dieser Kritik vgl. Roelcke (2008; 2012; 2013). Ebenfalls in einem größeren zeitlichen Zusammenhang wurde das wechselhafte Verhältnis der Disziplinen analysiert von Pantel (1993).

13 Die unversöhnliche Haltung gegenüber den Vertretern der Psychiatrie hatte höchstwahrscheinlich ihre Ursache in den Auseinandersetzungen an der Universität Heidelberg um die Etablierung der Psychosomatik. Dort hatten sich die Psychiater vehement gegen Mitscherlich (und Weizsäcker) gewandt. Vgl. Roelcke (1995).

14 Gerade, weil dies ein heikler Punkt war, legte er zugleich Wert darauf, nicht mit jenen Teilen der psychoanalytischen und psychologischen Theorie und Praxis assoziiert zu werden, die auf breite Ablehnung stießen. Zur prekären Stellung der Psychoanalyse in den ersten Nachkriegsjahren vgl. Kauders (2014: 161–202).

15 Vgl. hierzu den Beitrag von Maike Rotzoll in diesem Themenheft.

16 Siehe den Beitrag von Hofer in diesem Heft sowie Hofer (2019), Geisthövel & Hitzer (2019: 194).

17 Auf die darstellerischen Schwierigkeiten bei der Wissensvermittlung hatte Mitscherlich schon in seiner 1947 erschienenen Schrift über den Ursprung der Sucht verwiesen: „Der Mittelnde und der Leser müssen gleichermaßen wissen, daß das Mitgeteilte bei aller Ernsthaftigkeit, mit der es ausgesprochen wird, gleichzeitig aber auch nur ein ‚Wink‘, ein Hinweis bleibt, daß dahinter die irrationale Tiefe jedes wirklichen, d. h. wirkenden Seins sich andeutet.“ (Mitscherlich 1947: 24 f.).

18 Nicht nur die Redner von 1949 hatten diesen Eindruck. Der Psychoanalytiker Clemens de Boor (1920–2005), der Mitte der 1950er Jahre an die von Alexander Mitscherlich geleitete Psychosomatische Universitätsklinik Heidelberg wechselte, erinnerte sich später ebenfalls an die Hoffnung, dass der Kongress eine „Phase fruchtbaren Gedanken- und Erfahrungsaustausches“ in der Medizin eingeleitet habe und sich schon bald immer mehr Mediziner vom Nutzen der psychosomatischen Betrachtungsweise überzeugen ließen (de Boor 1958: 511).

19 Selbstkritischer hielt Meinertz auf dem gleichen Kongress der DGPT (1958) fest, dass „enthusiastische Betrachter“ aus den eigenen Reihen „ihre positive Einstellung so überspitzt“ hätten, „daß die mehr konservativen Kritiker geneigt sind zu sagen: da bleiben wir lieber bei der bewährten anatomischen oder allenfalls chemischen Grundlage der Erscheinungen.“ Meinertz (1958: 521).

20 1960 wurde Mitscherlich Mitglied der Deutschen Psychoanalytischen Vereinigung und Leiter des neu eröffneten Instituts und Ausbildungszentrums für Psychoanalyse und Psychosomatik (ab 1964 Sigmund-Freud-Institut) in Frankfurt am Main. Auskunft über Mitscherlichs Lehranalyse (1958/1959) bietet Holmes (2016: 141–143).

21 Vgl. Mitscherlich (1958/1983: S. 116 f., 127 f., Zitat: 130). Schon zuvor hatte Mitscherlich betont, dass der Krankheitsbegriff der naturwissenschaftlichen Heilkunde und der Psychosomatischen Medizin so grundverschieden seien, dass man nicht einfach die Bewertungsmaßstäbe für ihre Methodik von der einen auf die andere übertragen könne. Vgl. Mitscherlich (1946/1977: 91).

22 Er hatte schon 1949 gefordert, dass die psychosomatische Forschung „die beiderseitige Subjektivität von Arzt und Krankem nicht in ihren Aussagen verdecken möge“. Aussprache (1949: 81).

23 Derartige Untersuchungen hätten andernorts bereits „höchst beunruhigendes Material zum Vorschein gebracht“. Mitscherlich (1983e: 458).

24 Als „Zumutungen“ bezeichnete Mitscherlich die verfeinerten Probleme und komplizierteren Denkmodelle der Psychosomatischen Medizin sowie die Skepsis gegenüber dem Anspruch, „alles, was am und im Körper an kranken und gesunden Tagen geschieht, eines Tages durch Kausalzusammenhänge auf einer einzigen Wirkungsebene erklären zu können“. Mitscherlich (1966/1969b: 58f.).

25 Sein Verhältnis zur Soziologie wird näher erläutert von Nolte (2008).

26 Die fehlende empirische Basis von Mitscherlichs Aussagen zur Arzt-Patienten-Beziehung kritisierten auch Zeitgenossen; vgl. Pflanz (1962/1988: 24).

27 Es ist eine Randbemerkung wert, dass Mitscherlich hier einen Vorwurf umkehrte, den Martini 1949 der Medizinischen Anthropologie und damit auch der Psychosomatischen Medizin gemacht hatte: Er hatte ihr vorgehalten, wenn sie weiterhin „auf die Kausalität“ verzichte, werde sie „nicht zur Reformbewegung, sondern nur zur Sekte“. Martini (1949: 54).

28 Die Zeit erfordere „ein Denken, welches mehr Unsicherheit, mehr vorläufige Widersprüche erträgt“. Mitscherlich (1966/1969b: 60).

29 Dass die Beschränkung auf Kritik nicht ganz unproblematisch war, ahnte Mitscherlich wohl. So betonte er an zentralen Stellen seiner Texte, dass man – aufgrund der schwierigen Bedingungen, die man nicht selbst verschuldet habe – immer noch mehr „erahne“ als „wisse“ (Mitscherlich 1966/1969a: 33) oder dass man die speziellen methodischen Probleme kaum en détail schildern könne (Mitscherlich 1960: 36).

30 Man sollte diese gelegentlichen Diskussionen für Mitscherlichs Argumentationswandel jedoch nicht überschätzen. Martinis Forderungen und Angriffe waren meist lediglich eine mal mehr, mal weniger willkommene Gelegenheit zur öffentlichen Erwiderung.

31 Hoyer spricht von einer „Grauzone zwischen der Naturwissenschaft und der Geisteswissenschaft“. Hoyer (2008: 183).
